# Effects of Essential Oils from Zingiberaceae Plants on Root-Rot Disease of *Panax notoginseng*

**DOI:** 10.3390/molecules23051021

**Published:** 2018-04-26

**Authors:** Wu-Mei Sun, Yu-Nan Ma, Yan-Jiao Yin, Chuan-Jiao Chen, Fu-Rong Xu, Xian Dong, Yong-Xian Cheng

**Affiliations:** 1 College of Pharmaceutical Sciences, Yunnan University of Traditional Chinese Medicine, Kunming 650500, China; 14787473402@163.com (W.-M.S.); mayunan994727@163.com (Y.-N.M.); m15288241220@163.com (Y.-J.Y.); chenchuanjiao663@163.com (C.-J.C.); xfrong99@163.com (F.-R.X.); 2 Guangdong Key Laboratory for Genome Stability & Disease Prevention, School of Pharmaceutical Sciences, Shenzhen University Health Science Center, Shenzhen 518060, China

**Keywords:** Zingiberaceae, *Panax notoginseng*, root-rot disease, fungi, essential oils

## Abstract

Replanting obstacles of *Panax notoginseng* caused by complex factors, including pathogens, have received great attention. In this study, essential oils (EOs) from either *Alpinia officinarum* Hance or *Amomum tsao-ko* (Zingiberaceae) were found to inhibit the growth of *P. notoginseng*-associated pathogenic fungi in vitro. Subsequent GC-MS analysis revealed the chemical profiles of two plant derived EOs. Linalool and eucalyptol were found to be abundant in the EOs and tested for their antifungal activities. In addition, the synergistic effects of *A. tsao-ko* EOs and hymexazol were also examined. These findings suggested that Zingiberaceae EOs might be a good source for developing new green natural pesticides fighting against root-rot of *P*. *notoginseng*.

## 1. Introduction

*Panax notoginseng*, a well-known and valuable herb in China, is mainly cultivated around Wenshan in Yunnan Province, China [1]. Continuous cropping obstacles of *P. notoginseng* have received wide attention because of their threat to the yields and medicinal quality of the roots. Continuous cropping obstacles are a consequence of many complex factors, of which, root-rot disease caused by pathogenic fungal invasion cannot be ignored. Previous studies disclosed that *Fusarium oxysporum*, *Fusarium solani*, and *Cylindrocarpon destrutans* are closely related to the root-rot disease of *P. notoginseng* [2]. Therefore searching for antifungal agents toward these fungi might be beneficial for controlling root-rot and facilitate sustainable development of the *P. notoginseng* industry. In contrast to the disadvantages brought by chemical pesticides, botanical pesticides are relatively safe to health, friendly to the environment, which therefore have attracted great attention in the past years [3].

The family Zingiberaceae encompasses around 1500 plants. Many species in this family are important condiments and are widely used in the food industry as aromatic materials or spice additives. Polysaccharides, flavonoids, and EOs were documented from Zingiberaceae [4,5]. In fact, plants produce a large amount of secondary metabolites, many of which play an effective role in plants against pests and pathogens [6]. Inspired by a philosophy in traditional Chinese medicine that aromatic components generally suppress microorganisms, the EOs from four Zingiberaceae plants were extracted and examined for their antifungal properties against the pathogenic fungi, associated with the root-rot of *P. notoginseng*. In recent years, scholars in various countries have done much research on the composition analysis, antibacterial activity and other biological activities of EOs from Zingiberaceae plants, while their main antifungal components and antifungal mechanisms were not yet clear. Therefore, the specific active components and antifungal mechanism of EOs of Zingiberaceae need to be further explored and studied. The EO from *A. officinarum* could prevent food and aquatic products from spoil-aging, because it had obvious bacteriostatic and antifungal activity [7,8]. The *A. tsao-ko* EO had different degree inhibition to Gram-negative bacteria, Gram-positive bacteria and *Penicillium* [9,10]. 

In this study, the effects of essential oils from four kinds of Zingiberaceae plants on the main pathogenic fungi of *P. notoginseng* root-rot were studied, in order to increase the exploitation and utilization of essential oil resources from Zingiberaceae plants on the basis of existing research, and find a new biocontrol way to fight against *P. notoginseng* root-rot.

## 2. Results 

### 2.1. Inhibition of EOs from Zingiberaceae

**Figure 1:**
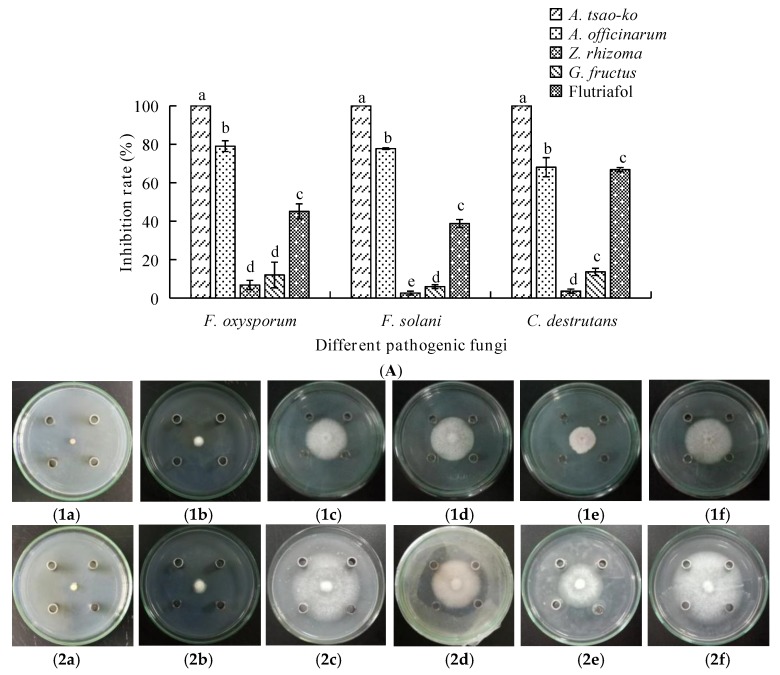

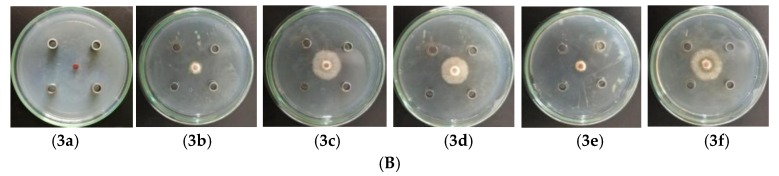
(**A**) The inhibitory rate of four EOs from Zingiberaceae on the growth of three fungi. (**B**) Fungi colony of the three pathogens treated by four EOs from Zingiberaceae. (**B1**) The colony diameter of *F*. *oxysporum* with different treatments. (**B2**) The colony diameter of *F*. *solani* with different treatments. (**B3**) The colony diameter of *C*. *destrutans* with different treatments. Besides, (**a**–**f**) standing for different treatments; (**a**) *A*. *tsao-ko* EO; (**b**) *A*. *officinarum* EO; (**c**) *Z*. *rhizoma* EO; (**d**) *G*. *fructus* EO; (**e**) flutriafol; (**f**) negative control.

As shown in Figure 1, the EOs from four different Zingiberaceae plants all exhibited inhibition against the three fungal strains *(*Figure 1B). Among them, the EOs from *A. tsao-ko* and *A. officinarum* were much stronger against the three fungi (Figure 1A). In detail, A. tsao-ko EO could completely inhibit *F. oxysporum*, *F. solani*, and *C. destrutans* at 50 mg/mL. The inhibitory effects of EO from *A. officinarum* appeared lower than those of *A. tsao-ko* EO against *F. oxysporum* (79.02%), *F. solani* (77.74%) and *C. destrutans* (68.08%), respectively. As for the activity of the EOs from *Z. rhizoma* and *G. fructus* against the three pathogenic fungi, their inhibitory effects were much weaker compared with *A. officinarum* and *A. tsao-ko*. With this, the antifungal properties of EOs from *A. tsao-ko* and *A. officinarum* against the pathogenic fungi were further investigated.

### 2.2. Determination of IC_50_ Values

To compare the antifungal activities of EOs between *A. tsao-ko* and *A. officinarum* aganist the three pathogenic fungal, the IC_50_ values were measured. As shown in Table 1, the IC_50_ values of *A. tsao-ko* EO for *F*. *oxysporum*, *F*. *solani*, and *C*. *destrutans* were 5.37 mg/mL, 55.42 mg/mL and 109.27 mg/mL, respectively. Whereas, the IC_50_ values of EO from *A. officinarum* for *F*. *oxysporum*, *F*. *solani*, and *C*. *destrutans* were 33.16 mg/mL, 54.78 mg/mL, and 64.13 mg/mL, respectively. Both EOs exhibited antifungal properties against the three fungi. Further we found that the inhibitory effects of *A. tsao-ko* and *A. Officinarum* against *F. oxysporum* were much better than those of *F. solani* and *C. destrutans*.

### 2.3. GC/MS Analysis of EOs

The EOs was obtained by hydrodistillation with a yield of 2.16% for *A. tsao-ko* and 0.54% for *A. officinarum*. The chemical composition of EOs was analyzed by GC/MS and the results were presented in Tables S1 and S2. 

There were 62 components in the EO of *A. tsao-ko*. Camphene (13.80%), zingiberene (13.18%), cis-citral (8.60%), eucalyptol (9.37%) and geranial (11.05%) were found to be the major compounds, accounting for 56.00%. Besides, 85 compounds were identified from *A. officinarum* EO. Linalool (20.25%) was the most abundant component. The other abundant compounds were found to be caryophyllene (12.80%), decyl ester (7.03%), and 1-decanol (5.02%), respectively.

### 2.4. The Inhibitory Effects of EO, Camphene, and Eucalyptol from A. tsao-ko

**Figure 2:**
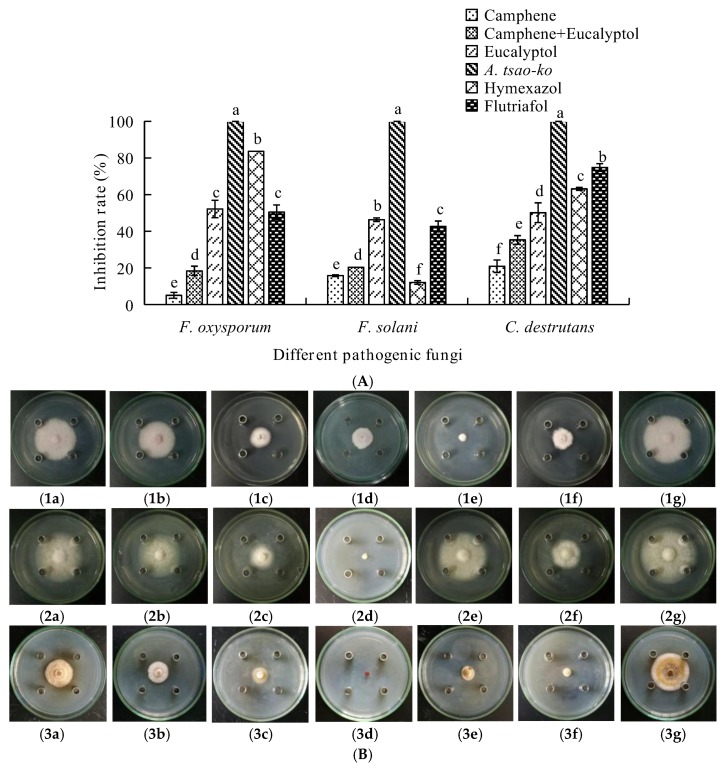
(**A**) The inhibitory rates of *A. tsao-ko* EO, camphene, eucalyptol, and camphene mixing with eucalyptol on the three fungi. (**B**) Fungi colony of the three pathogens by different treatments. (**B1**) The colony diameter of *F*. *oxysporum* with different treatments. (**B2**) The colony diameter of *F*. *solani* with different treatments. (**B3**) The colony diameter of *C*. *destrutans* with different treatments. Besides, (**a**–**f**) standing for different treatments; (**a**) camphene; (**b**) camphene mixing with eucalyptol; (**c**) eucalyptol; (**d**) *A. tsao-ko* EO; (**e**) hymexazol; (**f**) flutriafol; (**g**) negative control.

As presented in Figure 2A, the EO from *A. tsao-ko* exerted the strongest inhibition against the fungi with inhibition rates for *F*. *oxysporum*, *F*. *solani* and *C*. *destrutans* were all 100%. 

Eucalyptol showed moderate inhibition on mycelial growth with inhibition rates for *F. oxysporum*, *F. solani* and *C. destrutans* of 52.19%, 46.35%, and 50.13%, respectively (Figure 2B). However, the mixed compounds and camphene were less toxic to the three fungi, which were compared with the negative control.

### 2.5. Antifungal Properties of Linalool and Caryophyllene of EO from A. officinarum 

**Figure 3:**
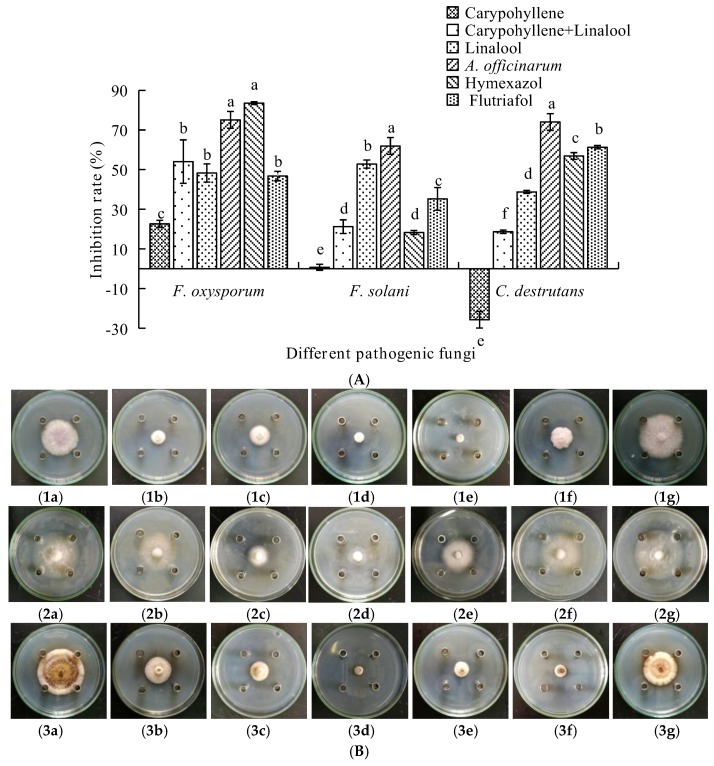
(**A**) The inhibitory rates of *A. officinarum* EO, caryophyllene, linalool, and caryophyllene mixing with linalool on the three pathogenic fungal. (**B**) Fungi colony of the three pathogens by different treatments. (**B1**) The colony diameter of *F*. *oxysporum* with different treatments. (**B2**) The colony diameter of *F*. *solani* with different treatments. (**B3**) The colony diameter of *C*. *destrutans* with different treatments. Besides, (**a**–**f**) standing for different treatments; (**a**) caryophyllene; (**b**) caryophyllene mixing with linalool; (**c**) linalool; (**d**) *A. officinarum*; (**e**) hymexazol; (**f**) flutriafol; (**g**) negative control.

The antifungal activities of linalool, caryophyllene, the mixture, and the EO from *A. officinarum* against the three fungi were examined (Figure 3). All the tested compounds showed notable antifungal activities (Figure 3B). The EO from *A. officinarum* was the most potent, with inhibition rates for *F*. *oxysporum*, *F*. *solani*, and *C*. *destrutans* of 75.09%, 61.91%, and 74.02%, respectively. 

The inhibitory activities of linalool was also remarkable with inhibition rates for *F*. *oxysporum*, *F*. *solani*, and *C*. *destrutans* of 48.28%, 52.80%, and 38.73%, respectively (Figure 3A). In contrast, the inhibitory activities of carypohyllene were less for *F*. *oxysporum* (22.61%) and *F*. *solani* (0.71%). It was surprising that caryophyllene was found to promote *C*. *destrutans* growth (26.10%).

### 2.6. Useful to Mix “Natural” and “Chemical” Fungicides

**Figure 4:**
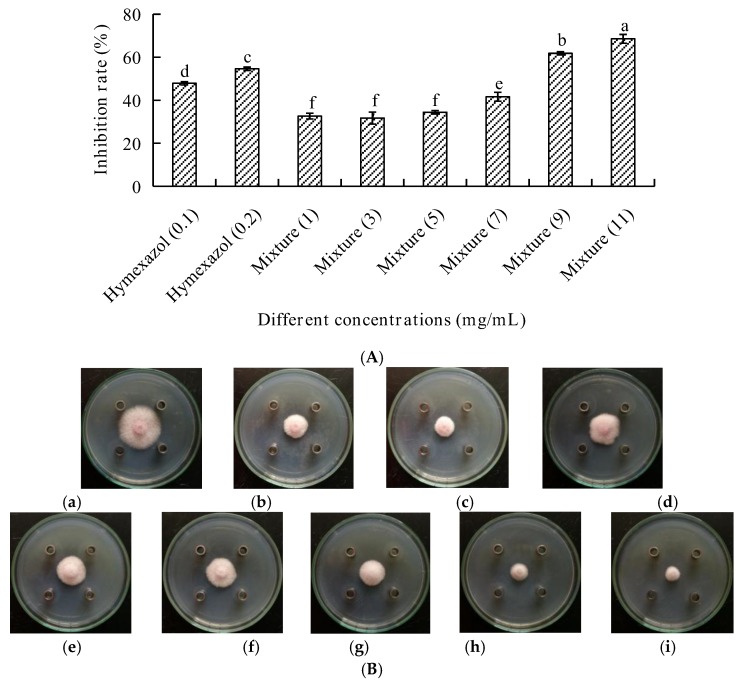
(**A**) The inhibitory rates of hymexazol and *A. tsao-ko* mixture on *F. oxysporum*. (**B**) The fungi colony of *F. oxysporum* by different treatments. Besides, (**a**–**f**) standing for different treatments. (**a**) negative control; (**b**) 0.1 mg/mL hymexazol; (**c**) 0.2 mg/mL hymexazol; (**d**) 0.1 mg/mL hymexazol and 0.9 mg/mL *A. tsao-ko* mixture; (**e**) 0.1 mg/mL hymexazol and 2.9 mg/mL *A. tsao-ko* mixture; (**f**) 0.1 mg/mL hymexazol and 4.9 mg/mL *A. tsao-ko* mixture; (**g**) 0.1 mg/mL hymexazol and 6.9 mg/mL *A. tsao-ko* mixture; (**h**) 0.1 mg/mL hymexazol and 8.9 mg/mL *A. tsao-ko* mixture; (**i**) 0.1 mg/mL hymexazol and 10.9 mg/mL *A. tsao-ko* mixture.

To observe the synergistic effects of natural EOs with chemical pesticide, the EO from *A. tsao-ko* mixed with hymexazol was examined for their inhibition against *F. oxysporum.* The results showed that different concentrations of EOs exerted different antifungal activities (Figure 4). When the concentration of the mixture (different concentrations of EO mixing with 0.1 mg/mL of hymexazol) was less than 7 mg/mL, the inhibition rate on *F. oxysporum* was less than 41.60% (Figure 4A). While the concentration of hymexazol was 0.2 mg/mL, the inhibition rate against *F. oxysporum* was 54.63%. In addition, when the concentration of the mixture (8.9 mg/mL of the EO mixing with 0.1 mg/mL of hymexazol) was 9 mg/mL, the antifungal effect was significant, and the inhibition rate against *F. oxysporum* was 61.81%, which inhibition rate was better than the positive (hymexazol of 0.2 mg/mL).

## 3. Discussion

Root-rot is a worldwide soil-borne disease, which can seriously damage many crops and medicinal plants and limit the continuous development of agriculture. Root-rot can occur in one- or two-year growth of *P. notoginseng*, whereas occurrence in three-year *P. notoginseng* is more severe. [11]. Previous studies have shown that *P. notoginseng* is a highly profitable medicinal crop. Farmers often blindly apply more fertilizer to control the root-rot in the hope of maximizing profits [12], which resulted in multiple chemical pesticide widely used in *P. notoginseng* planting. However, frequent use of chemical pesticide will lead to the salinization of the soil and the decrease or even loss of the protective effect of some biological control agents [13,14,15]. Chemical pesticides not only bring serious pollution and harm to ecology and environment but also have a bad effect on human health [16]. Hymexazol is a widely used chemical pesticide in the prevention and control of root-rot of *P. notoginseng*. However, their high mobility potential in the soil makes them problematic pesticides. Indeed, flutriafol is a potentially toxic chemical pesticide, which may disrupt fertility in women and affect the endocrine system [17]. *F. oxysporum*, *F. solani*, and *C. destrutans* are the causal pathogens of root-rot of *P. notoginseng*, which endangers *P. notoginseng* production. Although chemical fungicides are often used as the first defence against the fungal diseases. While the global contemporary trend has transferred to safer and environmental friendly methods to control these microbes and fungi [18]. Therefore, it is much significant to search for natural and potential antimicrobial agents to control the root-rot of *P. notoginseng*.

**Figure 5:**
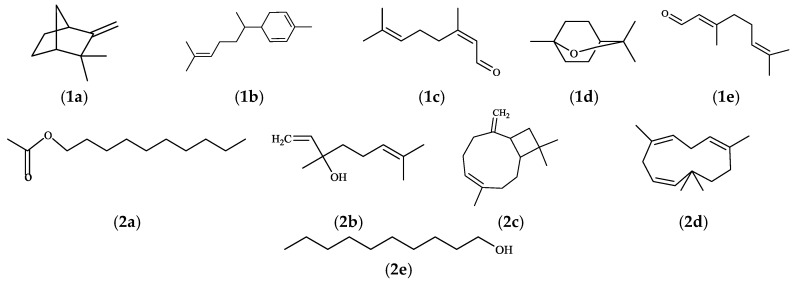
The chemical structures of principal components from *A. tsao-ko* EO (**1a**) camphene; (**1b**) zingiberene; (**1c**) *cis*-citral; (**1d**) eucalyptol; (**1e**) geranial; The chemical structures of principal components from *A. officinarum* EO (**2a**) decyl acetate; (**2b**) linalool; (**2c**) caryophyllene; (**2d**) 1,4,7-cycloundecatriene, 1,5,9,9-tetramethyl-Z,Z,Z-; (**2e**) 1-decanol.

The aim of this study was to investigate the potential antifungal effects of EOs from Zingiberaceae on root-rot of *P. notoginseng*. It was found that the inhibition rate of *A. tsao-ko* even reached up to 100%. Subsequently, 62 components were identified from the EO of *A. tsaoko* and camphene, zingberene, geranial, eucalyptol, and *cis*-citral were found to be abundant in the EO. The chemical structures of principal components from A. tsao-ko EO was shown in Figure 5. Previous studies identified 73 compounds from *A. tsao-ko* EOs, and the abundant compounds were eucalyptin, geraniol, and β*-p*-phenylbenzoic acid [19]. These differences might result from complex factors such as geographic regions, collection time, storage of material, and extraction methods. The EO from *A. officinarum* mainly contained linalool, caryophyllene, decylacetate, and 1-decanol accounting for 45.10% of the total EO. It was reported that EOs had antifungal activity, which could be attributed to the low-molecular-weight phenols, terpenes, and aldoketones [20]. These studies laid the foundations for our study because EOs from Zingiberaceae plants were also found to contain these components. From Figure 2, it could be seen that eucalyptol in *A. tsao-ko* EO exerted stronger antifungal effects on the three pathogenic fungal strains compared with the control treatment. Linalool in *A. officinarum* EO also had good antifungal effects against these pathogens (Figure 3). Therefore, it could be predicted that the antifungal activity of the EOs from *A. tsao-ko* and *A. officinarum* were related to the presence of eucalyptol and linalool. Thus it was probable that we could use linalool and eucalyptol as active substances to produce natural antifungal agents to fight against root-rot of *P. notoginseng.* In the present study, we also observed that antifungal activities of the EOs were the best with inhibition rate of *A. tsao-ko* EO to be 100% at the concentration of 50 mg/mL against the three pathogenic fungi. Due to the strong antifungal effect of *A. tsao-ko* EO on *F*. *oxysporum*, synergistic effects between *A. tsao-ko* EO with different concentrations and hymexazol (0.1 mg/mL) were observed. The inhibition rate of the mixture was 61.81% when the concentration was 9 mg/mL (8.9 mg/mL of *A. tsao-ko* EO mixing with 0.1 mg/mL of hymexazol). The inhibition rate was 7.18% higher than that 0.2 mg/mL of hymexazol. The results indicated that the EO could reduce the dosage of chemical pesticide used. These observations suggested that it is possible to use cocktail strategy by utilizing natural EOs to optimize a safer and more effective pesticide fighting against root-rot of *P. notoginseng*. It could be seen that the EOs can be used as a natural broad antifungal agent in agriculture, then the antifungal mechanism of the EOs remains to be further studied.

## 4. Materials and Methods 

### 4.1. Preparation of EOs

**Figure 6:**
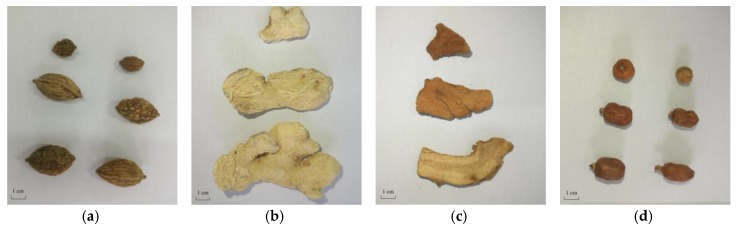
(**a**) *Amomum tsao-ko* (dried ripe fruit); (**b**) *Alpinia officinarum* (dry rhizoma); (**c**) *Zingiberis rhizoma* (dry rhizoma); (**d**) *Galangae fructus* (dried ripe fruit).

Four kinds of traditional Chinese medicinal materials *Amomum tsao-ko*, *Alpinia officinarum*, *Zingiberis rhizoma* and *Galangae fructus* (Figure 6) were purchased from Yunnan Jinfa Pharmaceutical Limited Company (Kunming, Yunnan of China). Medicinal parts of *A. tsao-ko* and *G. fructus* were the dried ripe fruit. *A. officinarum* and *Z. rhizoma* medicinal parts were dry rhizoma. The four Zingiberaceae plants were identified by one of our authors (Yong-Xian Cheng). EOs were prepared respectively from four Zingiberaceae plants by steam distillation for 7 h with 8-fold water (*v*/*w*). The EOs were collected and dried by sodium sulphate and then stored at −20 °C before use.

### 4.2. Fungal Strains

*F. oxysporum*, *F. solani* and *C. destructans* were obtained from Yunnan Agricultural University, China. The fungi were cultured on potato dextrose agar (PDA) medium at 28 °C in the microbiological incubator for 7 days.

### 4.3. Antifungal Determination of EOs by the Oxford Cup Method

The inhibitory effects of EOs were measured by the Oxford cup method [21]. EOs were dissolved in a mixture of solution (10/1000 DMSO and 1/1000 Tween-80) and mixed uniformly. Then they were filtered through 0.22 μm organic filter (Millipore, Kunming, Yunnan of China) and the final concentration was 50 mg/mL. PDA medium of 15 mL was poured into the dish. The mycelium block was obtained with a 5 mm diameter hole punch and placed in the middle of the Petri dish. Then the four Oxford cups were placed in the same distance around the fungi discs. The distance between the Oxford cup and the middle of Petri dish was 25 mm, then 200 μL of EOs solution was added into each oxford cup. A mixture of 10/1000 DMSO and 1/1000 Tween-80 solution was selected as a negative control, and 5 mg/mL of flutriafol was used as positive control. Each treatment was repeated four times. *F. oxysporum* and *F. solani* were cultured in the incubator at 28 °C for 5 days, with *C. destrutans* for 9 days. The radial growth (RG) of the fungi was determined by measuring the average of two perpendicular diameters. The growth inhibition rate was calculated as follows:

(1) $$\mathrm{Growth}\;\mathrm{inhibition}\;\mathrm{rate}=\frac{\mathrm{RG}\;\mathrm{of}\;\mathrm{negative}\;\mathrm{control}-\mathrm{RG}\;\mathrm{of}\;\mathrm{treated}\;\mathrm{sample}}{\mathrm{RG}\;\mathrm{of}\;\mathrm{negative}\;\mathrm{control}}\times 100\%$$

### 4.4. IC_50_ Determination of EOs

The IC_50_ values were determined by a described method [22]. Inhibition rates of EOs with more than 30% were selected for further IC_50_ measurement. EOs were dissolved in a mixture solution of 10/1000 DMSO and 1/1000 Tween-80, then they were diluted with 2-fold dilution method with a range of 1.172–600 mg/mL. The EOs were filtered through 0.22 µm organic filters (Millipore). To each tube, a mixture of EOs at different concentrations (20 µL) and a quarter PDA without agar (150 µL) were added to the cells of 96-well plates. Then a standardized suspension of the fungi (30 µL), which the cell concentration was 1 × 10^6^ CFU/mL, was added to each cell. The PDA of 150 µL without agar and 50 µL solution including 10/1000 DMSO and 1/1000 of Tween-80 were set as a negative control. Hymexazol was used as a positive control. Then the 96-well plates were incubated at 28 °C for 36 h. The level of growth inhibition was determined at 595 nm by using a microplate reader (Model 1510, Thermo, Shanghai, China).

### 4.5. GC/MS Analysis of EOs

The EOs from *A. tsao-ko* and *A. officinarum* of Zingiberaceae were analyzed by GC/MS. The GC apparatus was an Agilent Technologies instrument (Santa Clara, CA, USA) equipped with an HP-5MS capillary column (30 m × 0.25 mm, film thickness of 0.25 μm). The oven temperature was initially set at 50 °C for 2 min and then raised up to 130 °C (at a rate of 5 °C per min), subsequently 4 °C/min up to 190 °C, then 20 °C/min up to 220 °C, held for 5 min. The electron ionization was 70 eV. The detector and injector temperature was set at 250 °C and 230 °C, respectively. Helium was used as the carrier gas at a flow rate of 1.0 mL/min. The scanned mass range was *m*/*z* 30–550.

### 4.6. Antifungal Determination of EOs and Compounds from EOs 

Camphene and eucalyptol (purity: 99%), purchased from Shanghai Molbase Miological Technology Limited Corporation (Shanghai, China), are the main compounds in *A. tsao-ko* EO. Linalool and caryophyllene (purity: 98%) from *A. officinarum* EO were purchased from Shanghai Yuanye Biotechnology Limited Corporation (Shanghai, China). The compounds were dissolved in mixtures of 10/1000 DMSO and 1/1000 Tween 80, and the final concentration was 50 mg/mL. The inhibitory effects of compounds in EOs on the three fungal strains were studied in the same procedure according to the 2.2 method. Hymexazol (5 mg/mL) and flutriafol (5 mg/mL) were set as the positive controls. *F. oxysporum* and *F. solani* were cultured in the incubator at 28 °C for 5 days, with *C. destrutans* for 9 days. Then the diameter of colony was measured.

### 4.7. Synergism of A. tsao-ko EO and Hymexazol against F. oxysporum 

The synergistic effects of *A*. *tsao-ko* EO and hymexazol against *F*. *oxysporum* was observed as the same method described as the Oxford cup method*.* Hymexazol (0.2 mg/mL) was set as the positive control. The first mixture concentration was 1 mg/mL (0.1 mg/mL of hymexazol mixed with 0.9 mg/mL *A. tsao-ko* EO), and the second mixture concentration was 3 mg/mL (0.1 mg/mL of hymexazol mixed with 2.9 mg/mL *A. tsao-ko* EO). The remaining concentrations were configured in the same way. Then the final concentrations of the mixture were 1, 3, 5, 7, 9, 11 mg/mL, respectively. The effects of the mixture solutions on pathogens were carried out according to the Oxford cup method above. *F*. *oxysporum* was cultured in an incubator at 28 °C for 5 days, and the diameter of colony was measured.

### 4.8. Statistical Analysis

Statistical analysis was performed with SPSS Statistics 19.00 (Stanford University, Stanford, CA, USA, 1968) by using One Way ANOVA and Duncan’s multiple comparisons test.

## Figures and Tables

**Table 1:** IC_50_ (mg/mL) values of EOs from Zingiberaceae and positive control (hymexazol) against three fungal strains.

	*F. oxysporum*	*F. solani*	*C. destrutans*
*A. tsao-ko*	5.37	55.42	109.27
*A. officinarum*	33.16	54.78	64.13
Hymexazol	25.63	36.74	15.66

## Supplementary Materials

The following are available online, Chemical composition of the EOs of *A. tsao-ko*. Table S2: Chemical composition of the EOs of *A. officinarum*.
