# Supplementary material for: Effects of Essential Oils from Zingiberaceae Plants on Root-Rot Disease of Panax notoginseng
Source: Molecules. 2018 Apr 26;23(5):1021. doi: 10.3390/molecules23051021 (PMC6102565; doi:10.3390/molecules23051021)
Supplement: Supplementary file 1 [file molecules-23-01021-s001.pdf]

# Effects of essential oils from Zingiberaceae plants on root-rot disease of *Panax notoginseng*

**Wu-Mei Sun[1,2#], Yu-Nan Ma[1#], Yan-Jiao Yin [1,2], Chuan-Jiao Chen [1], Fu-Rong Xu [1], Xian Dong [1*] and Yong-Xian Cheng [1,2*]**

[1] College of Pharmaceutical Sciences, Yunnan University of Traditional Chinese Medicine, Kunming 650500, People's Republic of China; dongxian_1655129@163.com (X.D.).

[2] Guangdong Key Laboratory for Genome Stability & Disease Prevention, School of Pharmaceutical Sciences, Shenzhen University Health Science Center, Shenzhen, 518060, People's Republic of China; yxcheng@szu.edu.cn (Y.-X.C.)

**Table S1.** Chemical composition of the EOs of *A. tsao-ko*.

| NO. | Compound Name | Component RT | Relative content (%) |
|---|---|---|---|
| 1 | 2-heptanone | 5.82 | 0.03 |
| 2 | 2-heptanol | 6.04 | 0.22 |
| 3 | tricyclene | 6.62 | 0.24 |
| 4 | *α*-pinene | 6.94 | 3.68 |
| 5 | camphene | 7.35 | 13.80 |
| 6 | sabinene | 8.04 | 0.12 |
| 7 | (-)-*β*-pinene | 8.12 | 0.58 |
| 8 | 5-hepten-2-one, 6-methyl- | 8.42 | 0.55 |
| 9 | *β*-myrcene | 8.54 | 1.51 |
| 10 | octanal | 8.87 | 0.10 |
| 11 | *α*-phellandrene | 8.91 | 0.47 |
| 12 | 3-carene | 9.08 | 0.04 |
| 13 | *α*-terpinene | 9.27 | 0.03 |
| 14 | o-cymene | 9.51 | 0.11 |
| 15 | *β*-phellandrene | 9.64 | 8.48 |
| 16 | eucalyptol | 9.70 | 9.37 |
| 17 | 2-pyrrolidinone, 1-methyl- | 9.95 | 0.09 |
| 18 | 2-octenal, (*E*)- | 10.48 | 0.05 |
| 19 | *γ*-terpinene | 10.51 | 0.05 |
| 20 | terpinolene | 11.38 | 0.35 |
| 21 | 2-nonanone | 11.49 | 0.08 |
| 22 | linalool | 11.72 | 0.68 |
| 23 | (+)-2-bornanone | 13.03 | 0.23 |

| | | | |
|---|---|---|---|
| 24 | bicyclo[2.2.1]heptan-2-ol, 2,3,3-trimethyl- | 13.14 | 0.13 |
| 25 | 6-octenal, 3,7-dimethyl-, (*R*)- | 13.27 | 0.42 |
| 26 | *endo*-borneol | 13.39 | 0.06 |
| 27 | *endo*-borneol | 13.65 | 3.04 |
| 28 | (-)-terpinen-4-ol | 13.99 | 0.27 |
| 29 | isogeranial | 14.13 | 0.21 |
| 30 | $\alpha$-terpineol | 14.37 | 1.82 |
| 31 | myrtenal | 14.54 | 0.17 |
| 32 | 3-isopropylbenzaldehyde | 15.10 | 0.03 |
| 33 | citronellol | 15.42 | 0.67 |
| 34 | benzene, 2-methoxy-4-methyl-1-(1-methylethyl)- | 15.62 | 0.07 |
| 35 | *cis*-citral | 15.80 | 8.60 |
| 36 | geraniol | 16.15 | 1.31 |
| 37 | geranial | 16.63 | 11.05 |
| 38 | ethanone, 1-(2-hydroxy-5-methylphenyl)- | 16.82 | 0.04 |
| 39 | l-bornyl acetate | 17.06 | 0.09 |
| 40 | thymol | 17.20 | 0.10 |
| 41 | 2-undecanone | 17.23 | 0.19 |
| 42 | $\alpha$-copaene | 19.57 | 0.12 |
| 43 | acetic acid lavandulyl ester | 19.73 | 0.15 |
| 44 | $\beta$-elemene | 20.05 | 0.32 |
| 45 | (*1R*,*5R*)-2-methyl-5-((*R*)-6-methylhept-5-en-2-yl)bicyclo[3.1.0]hex-2-ene | 20.45 | 0.05 |
| 46 | caryophyllene | 20.92 | 0.07 |
| 47 | (*1S*,*5S*)-4-methylene-1-((*R*)-6-methylhept-5-en-2-yl)bicyclo[3.1.0]hexane | 22.08 | 0.18 |
| 48 | sesquisabinene | 22.28 | 0.08 |
| 49 | $\alpha$-curcumene | 23.01 | 2.56 |
| 50 | zingiberence | 23.47 | 13.18 |
| 51 | (+)-*epi*-bicyclosesquiphellandrene | 24.07 | 0.13 |
| 52 | $\beta$-sesquiphellandrence | 24.50 | 4.45 |
| 53 | (*E*)-1-methyl-4-(6-methylhept-5-en-2-ylidene)cyclohex-1-ene | 24.79 | 0.09 |
| 54 | elemol | 25.45 | 0.71 |
| 55 | *trans*-sesquisabinene hydrate | 25.61 | 0.19 |
| 56 | (1E,4E)-germacrene | 25.75 | 0.19 |
| 57 | (E)-nerolidol | 25.99 | 0.40 |
| 58 | *trans*-sesquisabinene hydrate | 27.04 | 0.54 |

| | | | |
|---|---|---|---|
| 59 | (*1R*,*4R*)-1-methyl-4-(6-methylhept-5-en-2-yl)cyclohex-2-enol | 28.00 | 0.82 |
| 60 | *β*-eudesmol | 29.47 | 0.38 |
| 61 | 3-cyclohexen-1-ol, 1-(1,5-dimethyl-4-hexenyl)-4-methyl- | 30.34 | 0.07 |
| 62 | phenol, 5-(1,5-dimethyl-4-hexenyl)-2-methyl-,(*R*)- | 33.91 | 0.02 |

**Table S2.** Chemical composition of the EOs of *A. officinarum.*

| NO. | Compound Name | Component RT | Relative content (%) |
|---|---|---|---|
| 1 | heptanal | 6.08 | 0.05 |
| 2 | *α*-pinene | 6.94 | 1.49 |
| 3 | camphene | 7.34 | 0.30 |
| 4 | benzaldehyde | 7.67 | 0.08 |
| 5 | (-)-*β*-pinene | 8.13 | 4.67 |
| 6 | 3-heptanone | 8.20 | 0.01 |
| 7 | sulcatone | 8.43 | 0.03 |
| 8 | *β*-myrcene | 8.54 | 1.30 |
| 9 | octanal | 8.87 | 0.23 |
| 10 | *α*-phellandrene | 8.91 | 0.15 |
| 11 | 3-carene | 9.08 | 0.06 |
| 12 | acetic acid, hexyl ester | 9.19 | 0.01 |
| 13 | isoterpinolene | 9.27 | 0.02 |
| 14 | o-cymene | 9.51 | 0.27 |
| 15 | *β*-phellandrene | 9.63 | 0.96 |
| 16 | eucalyptol | 9.69 | 0.65 |
| 17 | *trans*-*β*-ocimene | 9.90 | 0.03 |
| 18 | 2-pyrrolidinone, 1-methyl- | 9.95 | 0.08 |
| 19 | *β*-ocimene | 10.20 | 0.08 |
| 20 | γ-terpinene | 10.51 | 0.05 |
| 21 | acetophenone | 10.73 | 0.02 |

| | | | |
|---|---|---|---|
| 22 | 1-octanol | 10.85 | 0.22 |
| 23 | *trans*-linalool oxide (furanoid) | 10.92 | 0.30 |
| 24 | linalool | 11.77 | 20.25 |
| 25 | fenchol | 12.14 | 0.08 |
| 26 | (*E*)-4,8-dimethylnona-1,3,7-triene | 12.21 | 0.53 |
| 27 | (+)-2-bornanone | 13.03 | 0.19 |
| 28 | cycloprop[a]indene, 1,1a,6,6a-tetrahydro- | 13.22 | 0.07 |
| 29 | benzenepropanal | 13.55 | 0.13 |
| 30 | *endo*-borneol | 13.65 | 0.37 |
| 31 | 1-nonanol | 13.79 | 0.02 |
| 32 | (-)-terpinen-4-ol | 13.99 | 0.18 |
| 33 | *α*-terpineol | 14.37 | 1.12 |
| 34 | 4-decenal, (*E*)- | 14.45 | 0.90 |
| 35 | decanal | 14.78 | 1.06 |
| 36 | acetic acid, octyl ester | 14.95 | 0.90 |
| 37 | citronellol | 15.41 | 0.54 |
| 38 | benzene, 2-methoxy-4-methyl-1-(1-methylethyl)- | 15.62 | 0.12 |
| 39 | neral | 15.78 | 0.09 |
| 40 | 2-butanone, 4-phenyl- | 15.86 | 0.12 |
| 41 | 10-dodecenol | 15.99 | 0.06 |
| 42 | geraniol | 16.15 | 0.67 |
| 43 | 5-decen-1-ol, (*E*)- | 16.22 | 1.92 |
| 44 | 2-decenal, (*E*)- | 16.34 | 0.16 |
| 45 | 1-decanol | 16.64 | 5.02 |
| 46 | bornyl acetate | 17.05 | 0.10 |
| 47 | l-bornyl acetate | 17.05 | 0.10 |
| 48 | thymol | 17.21 | 0.07 |
| 49 | acetic acid, nonyl ester | 17.69 | 0.05 |
| 50 | *trans*-geranic acid methyl ester | 18.05 | 0.08 |
| 51 | (＋/－)-*ς*-elemene | 18.46 | 0.01 |
| 52 | citronellol acetate | 18.85 | 0.15 |
| 53 | 3-phenyl-1-propanol, acetate | 19.36 | 0.57 |
| 54 | *α*-copaene | 19.57 | 0.57 |
| 55 | (*Z*)-dec-4-en-1-yl propyl carbonate | 19.90 | 3.70 |
| 56 | benzene propanol, *α*-methyl-, acetate | 20.11 | 0.16 |
| 57 | decyl acetate | 20.56 | 7.03 |
| 58 | caryophyllene | 20.95 | 12.79 |
| 59 | (+)-*epi*-bicy closesquiphel landrene | 21.23 | 0.05 |

| | | | |
|---|---|---|---|
| 60 | *γ*-elemene | 21.35 | 0.04 |
| 61 | 1,4,7,-cycloundecatriene, 1,5,9,9-tetramethyl-,Z,Z,Z- | 22.05 | 5.11 |
| 62 | 8-dodecenol | 22.16 | 0.11 |
| 63 | ethyi cinnamate | 22.37 | 0.78 |
| 64 | 1-dodecanol | 22.64 | 0.11 |
| 65 | (+)-*γ*-cadinene | 22.81 | 0.29 |
| 66 | *α*-curcumene | 23.00 | 2.40 |
| 67 | (+)-*β*-selinene | 23.17 | 0.19 |
| 68 | zingiberene | 23.45 | 0.84 |
| 69 | pentadecane | 23.56 | 0.57 |
| 70 | aciphyllene | 23.60 | 0.51 |
| 71 | aromandendrene | 23.91 | 0.40 |
| 72 | (+)-*γ*-cadinene | 24.15 | 0.35 |
| 73 | 1-isopropyl-4,7-dimethyl-1,2,3,5,6,8a- | 24.49 | 1.00 |
| 74 | (+)-valencene | 24.92 | 0.11 |
| 75 | *α*-calacorene | 25.22 | 0.10 |
| 76 | (1E,4E)-germaccrenceb | 25.75 | 0.08 |
| 77 | nerolidol | 26.01 | 4.14 |
| 78 | caryophyllene oxide | 26.77 | 2.66 |
| 79 | guaiol | 27.37 | 0.23 |
| 80 | (*1S,3aS,4S,5S,7aR,8R*)-5-isopropyl-1,7a-dimethyloctahydro-1h-1,4-methanoinden-8-ol | 27.61 | 0.20 |
| 81 | n-butyl cinnamate | 28.55 | 0.11 |
| 82 | di-*epi*-1,10-cubenol | 28.60 | 0.17 |
| 83 | farnesol | 32.61 | 0.37 |
| 84 | ethyl *p*-methoxycinnamate | 34.01 | 0.06 |
| 85 | trifluoroacetyl-lavandulol | 38.00 | 0.03 |
